# Microstructure and Properties of Pressureless-Sintered Zirconium Carbide Ceramics with MoSi_2_ Addition

**DOI:** 10.3390/ma17092155

**Published:** 2024-05-05

**Authors:** Xiuzheng Chen, Haibo Wu, Huan Liu, Yitian Yang, Bingbing Pei, Jianshen Han, Zehua Liu, Xishi Wu, Zhengren Huang

**Affiliations:** 1 School of Material Science and Chemical Engineering, Ningbo University, Ningbo 315211, China; chenxiuzheng@nimte.ac.cn; 2 Ningbo Institute of Materials Technology and Engineering, Chinese Academy of Sciences, Ningbo 315201, China; liuhuan@nimte.ac.cn (H.L.); yangyitian@nimte.ac.cn (Y.Y.); peibingbing@nimte.ac.cn (B.P.); hanjianshen@nimte.ac.cn (J.H.); liuzehua@nimte.ac.cn (Z.L.); wuxishi@nimte.ac.cn (X.W.)

**Keywords:** pressureless sintering, ZrC, mechanical properties, thermal properties

## Abstract

Zirconium carbide (ZrC) ceramics have a high melting point, low neutron absorption cross section, and excellent resistance to the impact of fission products and are considered to be one of the best candidate materials for fourth-generation nuclear energy systems. ZrC ceramics with a high relative density of 99.1% were successfully prepared via pressureless sintering using a small amount of MoSi_2_ as an additive. The influence of the MoSi_2_ content on the densification behavior, microstructure, mechanical properties, and thermal properties of ZrC ceramics was systematically investigated. The results show that the densification of ZrC was significantly enhanced by the introduction of MoSi_2_ due to the formation of a liquid phase during sintering. In addition, the ZrC grains were refined due to the pinning effect of the generated silicon carbide. The flexural strength and Vickers hardness of ZrC ceramics with 2.5 vol% MoSi_2_ sintered at 1850 °C were 408 ± 12 MPa and 17.1 GPa, respectively, which were approximately 30% and 10% higher compared to the samples without the addition of MoSi_2_. The improved mechanical properties were mainly attributed to the high relative density (99.1%) and refined microstructure.

## 1. Introduction

With the continuous development of industrial society and the increasing energy demand, as well as the increasingly serious effects of climate change, energy transformation and cleanliness have become particularly important. Nuclear energy, as one of the representatives of clean energy, has always been highly anticipated by people. From the 1950s to now, significant progress has been made in nuclear energy research and a series of breakthroughs have been achieved. However, there are still some challenges, such as inefficient use, with less than 1% of uranium resources effectively utilized; a large amount of nuclear waste generated with a long radioactive lifetime, making spent fuel disposal quite challenging; and an urgent need to strengthen the safety of nuclear energy systems [1]. In response to the current energy and environmental crisis and shortcomings in existing nuclear energy systems, countries have accelerated the development of advanced nuclear energy systems, including fourth-generation fission energy systems, nuclear fusion energy systems, and accelerator-driven advanced nuclear energy systems [2].

Compared with the current commercial mainstream second- and third-generation nuclear energy systems, fourth-generation nuclear power plants have comprehensively improved safety and economy sustainability, among other aspects. However, they also lead to more stringent requirements for the performance materials used in service conditions, including service temperature, neutron irradiation resistance, and corrosion resistance [3]. At present, there are two main research directions for these service materials: metal materials and ceramic materials. Compared with metals, ceramics possess higher melting points better mechanical properties high-temperature stability thus having great potential applications in advanced nuclear energy system materials [4].

Among many candidate ceramic materials, ZrC material has the advantages of the highest melting point, a low neutron absorption cross-section, and high irradiation stability and is considered to be one of the most promising candidate materials in advanced nuclear energy systems [5], as it can be used to make nuclear fuel cladding materials and inert matrix fuel materials [6,7,8]. However, ZrC ceramics are difficult to sinter densely due to their covalent bonds and low diffusion coefficients [8]. High-temperature and pressure-assisted techniques are often required to achieve dense sintered bodies. Spivak et al. [9] obtained ZrC with a density of about 94% by hot-press sintering at 20 MPa and holding at 2600 °C for 30 min. Zhao et al. [10] prepared dense ZrC-ZrB2 ceramics from ZrC and LaB6 by reactive-hot-press sintering at a temperature of 1900 °C and a pressure of 25 MPa. The in situ generation of layered structures can significantly improve the mechanical properties of materials. Sciti et al. [11,12] studied the SPS sintering of ZrC, and the relative density of ZrC reached about 98% when sintered at a pressure of 65 MPa and a sintering temperature of 2100 °C for 3 min. Gendre et al. [13] prepared dense ZrC ceramics with a relative density of 98% using the SPS sintering technique at sintering temperatures above 2000 °C and pressures greater than 50 MPa. However, high-temperature and high-pressure sintering methods limit the engineering applications of ZrC ceramics.

Pressureless sintering has the advantages of simplicity and convenience in preparing large-size samples. Previous studies have shown that the pressureless densification of ZrC with a relative density of 97–98% requires the high sintering temperature of 2400 °C to 2600 °C [14,15]. On the other hand, adding sintering additives is a common method for compacting sintering without pressure, but the addition of sintering additives will have a certain effect on the intrinsic properties of materials. From the background of the application in the field of nuclear energy, it is necessary to select a sintering agent with a small neutron absorption cross-section. MoSi_2_ has a low neutron absorption cross-section and is a good sintering agent object. Moreover, the introduction of MoSi_2_ changes the densification process from solid-phase sintering without additives to liquid-phase sintering of silicon-containing compounds, promotes grain rearrangement and material transfer in the sintering process, and accelerates the densification process. In addition, MoSi_2_ material itself has a high melting point (~2050 °C), good high-temperature mechanical and thermal properties, excellent oxidation resistance, and corrosion resistance, and is a very promising high-temperature material. Silvestroni et al. [16] found that ZrC-based composite ceramics doped with 20 vol% MoSi_2_ could be sintered to a relative density of 96.5% at 1950 °C via pressureless sintering. However, an excessive amount of MoSi_2_ or a high sintering temperature can negatively affect the mechanical properties. Therefore, it is necessary to study the densification of ZrC ceramics with low-dosage sintering additives.

In the present work, ZrC ceramics were pressureless-densified at 1750 °C to 1950 °C, with MoSi_2_ addition ranging from 0 to 5 vol%. The effects of MoSi_2_ content on the densification behavior, microstructure, mechanical properties, and thermal properties of ZrC ceramics were systematically investigated.

## 2. Materials and Methods

### 2.1. Raw Materials

**Figure 1:**
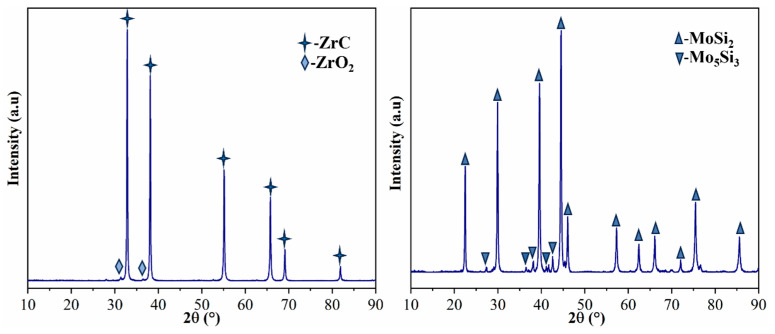
XRD pattern of powder.

**Figure 2:**
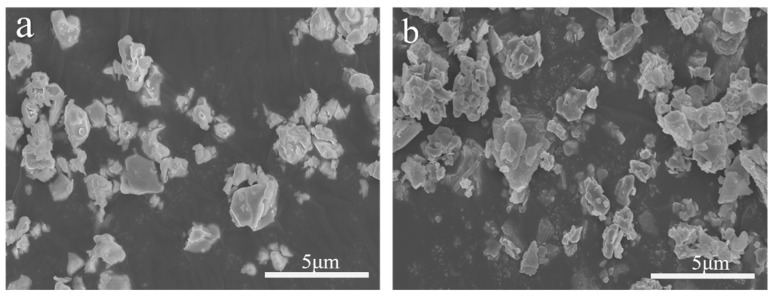
SEM powder morphology (**a**) ZrC; (**b**) MoSi_2_.

Commercial ZrC powder and MoSi_2_ powder were selected as starting materials; ZrC powder was purchased from Guangzhou Metallurgical Co., LTD. (Guangzhou, China); MoSi_2_ was purchased from Suzhou Bairui New Materials Co., LTD. (Suzhou, China). The main characteristics are summarized in Table 1. The information in Table 1 came from the raw material supplier. Figure 1 shows the XRD pattern of the original powder. ZrC powder contains a small amount of ZrO_2_, and MoSi_2_ powder contains a small amount of Mo_5_Si_3_, which may be related to the preparation method of the powder. The powder morphology is shown in Figure 2, and it can be seen that the powder particles are fine and uniform.

### 2.2. Preparation of the Samples

First, a certain volume ratio of ZrC and MoSi_2_ powder was weighed on the analytical balance and added to the polyurethane ball mill tank. Then, we added 1% of the total mixture mass PVB (polyvinyl butyral) as the binder, 5mm diameter of ZrO_2_ ball as the grinding medium, and the ball material ratio of 3:1, with an appropriate amount of anhydrous ethanol as the dispersion medium. The total volume did not exceed 1/2 of the mixing tank to ensure the uniformity of the mixing powder. After ball milling, the obtained slurry was put into an electric blast drying oven and dried for 2 h at a constant temperature of 60 °C. After grinding, the mixed powder of ZrC and MoSi_2_ was obtained through a 120-mesh screen. The mixed powder was put into the dry-pressing mold of the required shape, formed, and held under uniaxial pressure of 30 MPa for 30 s, and then treated under cold isostatic pressure at 200 MPa for 3 min to obtain the ceramic blank for sintering. Subsequently, the unpacked blanks were put into a graphite crucible in a vacuum horizontal sintering furnace to complete the sintering. Under vacuum, the temperature was increased to 600 °C at a rate of 4 °C/min and held for 0.5 h to remove the PVB added in the initial powder, which was thermally degraded and volatilized from the embryo at high temperature. Afterward, the temperature was increased to 1200 °C at a rate of 6 °C/min for 0.5 h to realize the pre-sintering of the sample. We continued to increase the temperature rate of 6 °C/min to 1500 °C, holding for 0.5 h, and then flowing argon gas was injected, followed by 3 °C/min rate of heating to the target temperature of 1750 °C~1950 °C and holding for 1 h.

**Figure 3:**
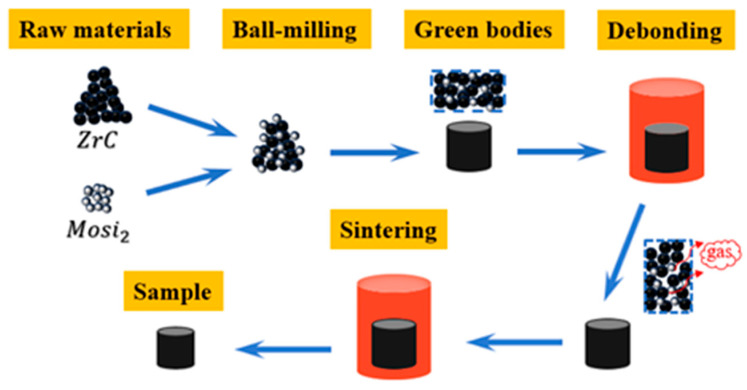
Preparation flowchart.

The preparation flowchart is shown in Figure 3.

The composition of different formula numbers is shown in Table 2.

### 2.3. Characterization of the Samples

The density and porosity of sintered samples were measured according to Archimedes’ principle. The relative density (RD) is the ratio of the density to the theoretical density, where the theoretical density was calculated using the mixing rule. X-ray diffraction (ADVANCED8, Burkle, Bad Bellingen, Germany) with Cu Ka radiation (k = 1.54056 Å) was used to identify the crystalline phase of the bulk samples, and the raw powder composition was analyzed. The ceramic samples’ bending strength and modulus of elasticity were measured using the three-point bending method on a universal material-testing machine (Z030TE+TEE, ZVIK, Frankfurt, Germany). The samples to be tested were machined into standard test strips of 3 mm × 4 mm × 36 mm and were polished with 5 μm diamond and chamfered on one side to eliminate the residual stresses introduced during the machining of the samples. The span was 30 mm; the loading speed was set to 0.5 mm/min. After spraying carbon on the surface of the samples, the thermal conductivity at room temperature was measured using a laser thermal conductivity meter (LFA467, NETZSCH, Hanau, Germany). The sample size was ø12.7 mm × 2 mm. Scanning electron microscopy (SEM, S-8230, Hitachi, Tokyo, Japan) was used to analyze the cross-section and polished surface (0.5 μm diamond ground etched with hydrofluoric acid for 4 h) of the samples. Particle-size statistics were performed using electron backscattering diffraction (EBSD) (SEM, G300, Zeiss, Oberkochen, Germany). The Vickers hardness and fracture toughness of the sample were measured by a Vickers hardness tester (VH3300, Wilson, Chicago, IL, USA) with a load of 5 Kg and residence time of 10 s.

**Figure 4:**
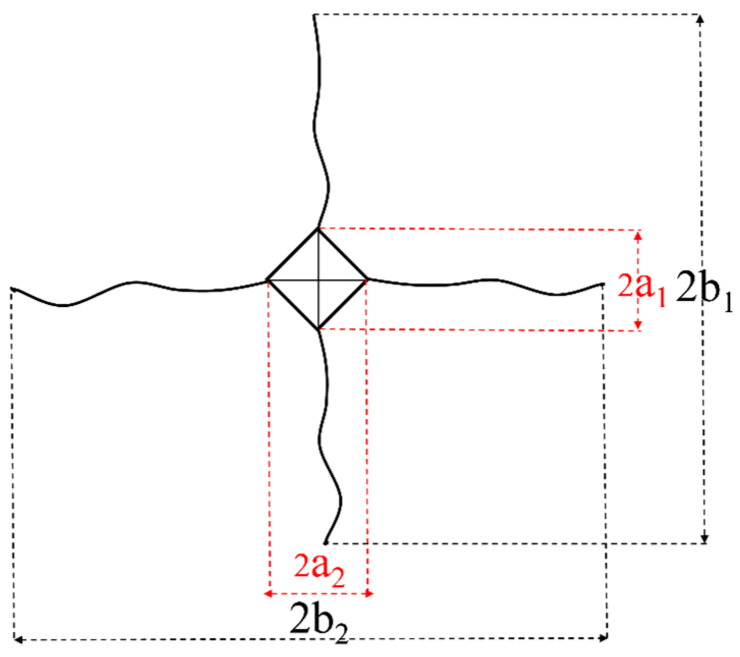
Diagram for calculating fracture toughness of material via indentation method.

The fracture toughness of the samples was measured using the indentation method. The method of calculating the fracture toughness of an object was as follows [17]:

(1) $$b=\left(b_{1}+b_{2}\right)/2$$

b_1_ and b_2_ are diagonal crack lengths, as shown in Figure 4, and b is the crack half-length

(2) $$K_{IC}=0.026\times (E^{\frac{1}{2}}P^{\frac{1}{2}}a/b^{\frac{3}{2}})$$

where K_IC_ is fracture toughness (Pa·m^1/2^); E is the elastic modulus (GPa); P is the test load (N); a is the diagonal half-length of the indentation (m); b is the crack half-length (m).

## 3. Results and Discussion

### 3.1. Densification Behavior of the ZrC Ceramics

**Figure 5:**
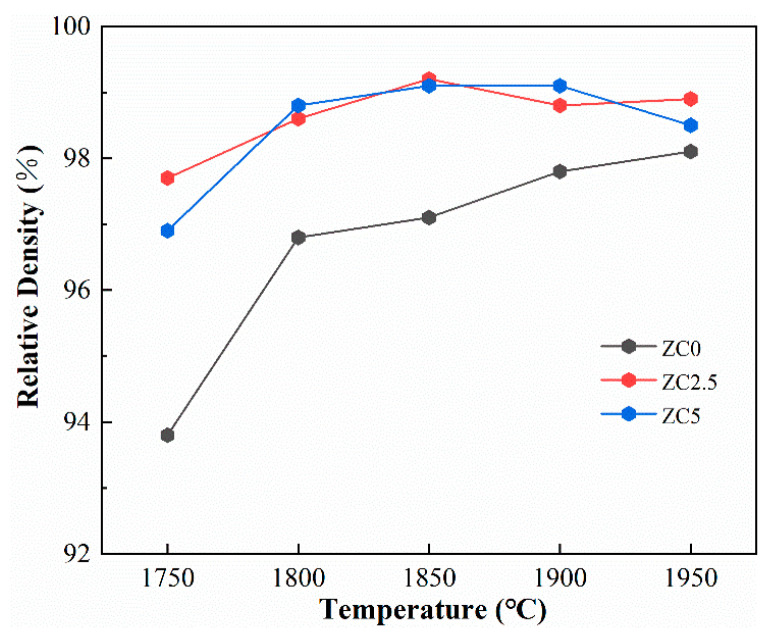
The relative density as a function of sintering temperature for ZrC samples.

Figure 5 illustrates the relative density of the resulting samples as a function of the temperature. The relative density increases gradually with increasing sintering temperature. The increase in temperature increases the sintering driving force, accelerates the mass transfer rate, and increases the relative density. ZrC without MoSi_2_ can be densified above 1800 °C. However, obtaining monolithic zirconium carbide ceramics with such high relative densities via pressureless sintering is often challenging.

The delicate and uniform initial particle size of zirconium carbide (500 nm) may be one of the reasons for the densification during sintering. The tiny particles shorten the diffusion distance of atoms during the sintering process, allowing the ceramic to achieve high relative density before significant grain growth occurs [18]. Moreover, the uniform particle-size distribution effectively inhibits grain coarsening and promotes sintering. In addition, zirconium oxide in the initial powder forms defective zirconium carbide compounds with ZrC. Oxygen in the zirconia diffuses into the ZrC lattice, leading to increased diffusion and densification, which may be another contributing factor [19].

Moreover, the introduction of MoSi_2_ significantly improved the sintering performance of zirconium carbide ceramics and reduced the densification temperature, and the influence of 2.5 vol% and 5 vol% MoSi_2_ additions on the density was similar. Due to the liquid phase formed by the reaction of MoSi_2_ and ZrC in the sintering process, the material transfer rate was improved. At a sintering temperature of 1850 °C, the relative density of a sample with 2.5 vol% MoSi_2_ content was as high as 99.1%.

### 3.2. Chemical Composition and Structure Analysis

**Figure 6:**
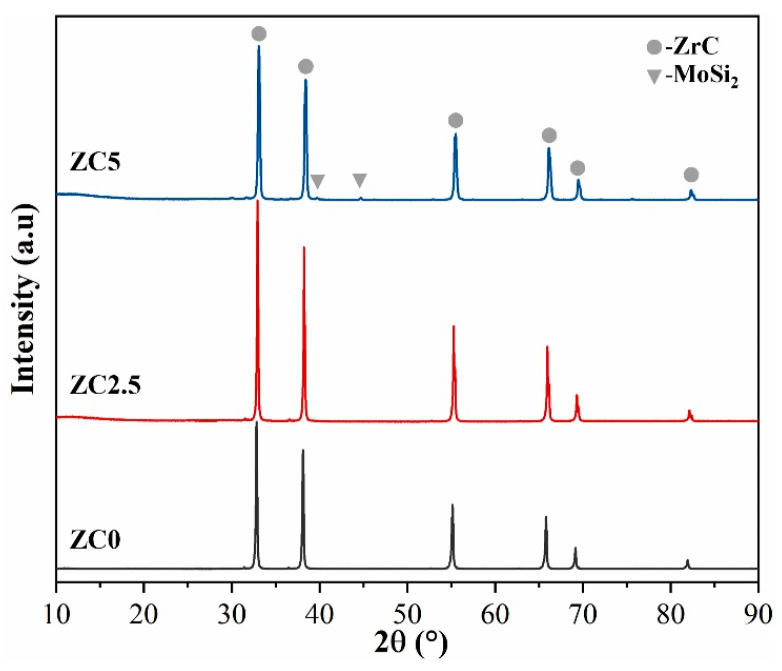
The XRD spectrum of ZrC ceramics sintered at 1850 °C.

**Figure 7:**
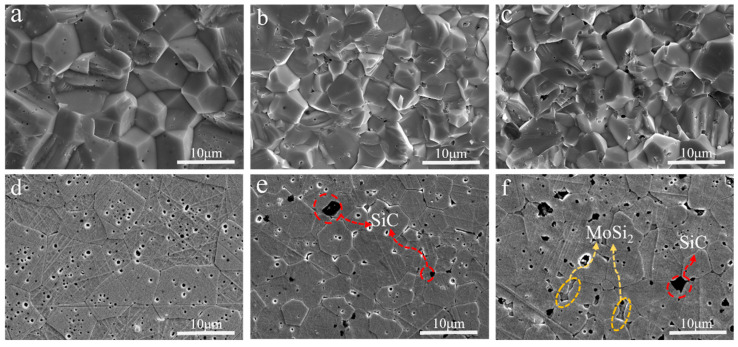
SEM images of the section and polished surface of the ZrC ceramics sintered at 1850 °C; (**a**–**c**) is a separate section of ZC0, ZC2.5, and ZC5; (**d**–**f**) is separately polished surface of ZC0, ZC2.5, and ZC5.

**Figure 8:**
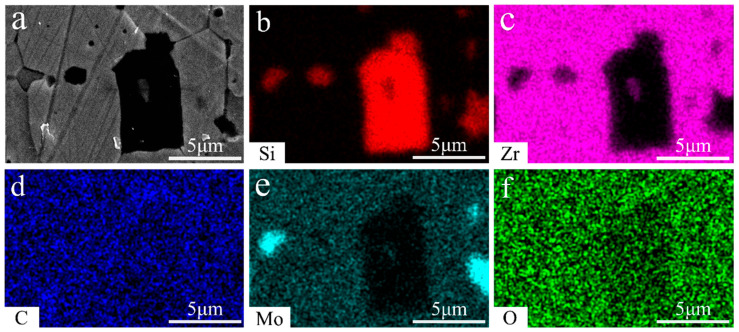
SEM images of the polished surface of the ZrC ceramics (ZC5): (**a**) high-magnification image; (**b**–**f**) the separate distribution of elements Si, Zr, C, Mo, O.

The XRD pattern of block ZrC ceramics is shown in Figure 6. Only peaks corresponding to ZrC and MoSi_2_ are observed and nothing else is found. No MoSi_2_ peaks were detected in ZC2.5 due to the relatively small amount of MoSi_2_ added (only 2.5 vol%), the reaction consumption caused by reaction with ZrC during sintering, and the liquid-phase volatilization under sintering, resulting in a further reduction in MoSi_2_ in the sample. However, MoSi_2_ peaks appeared in ZC5. In addition, no SiC phase (formed by reaction, as shown in Figure 7 and Figure 8) was found in the XRD spectra. Due to the low addition of MoSi_2_, the generated SiC content was low and did not reach the minimum detection line of XRD content, so the newly formed phase could not be directly detected via XRD.

The microstructure of ZrC ceramics is shown in Figure 7. The cross-sectional electron microscope image reveals that the fracture mode of the sample consists of a combination of trans-crystalline fracture and pre-fracture. However, with an increase in MoSi_2_ content, there is a higher proportion of trans-crystalline fracture, indicating enhanced grain boundary bonding strength due to the introduction of MoSi_2_. Figure 7d–f display the electron microscope images after hydrofluoric acid etching. It can be observed that ZrC ceramics exhibit a high relative density overall, with only a small amount of confined pores due to grain boundaries moving faster than the pore movement rate. This phenomenon is more evident in Figure 7d. In addition, Figure 7e,f also show new phases, which were analyzed via EDS surface scanning, as shown in Figure 8. The analytical results indicate that the black phase is SiC, the dark-gray phase is MoSi_2_, and the gray phase is ZrC, and these annotations are shown in Figure 7, accordingly. The generation of SiC during sintering via the introduction of MoSi_2_ is in agreement with the findings in reference [16]. In addition, it can be observed that MoSi_2_ was in a liquid state during sintering and usually appeared in narrow or irregular forms at the grain boundaries of zirconia.

However, MoSi_2_ shows a melting point of more than 2000 °C [20], which exceeds our sintering temperature (maximum sintering temperature of 1950 °C). However, the presence of carbon allows the formation of liquid phases in the C-Mo-Si system in arbitrary proportions [21]. ZrC is prone to non-stoichiometric ratios [22] and inevitably contains traces of free carbon in the starting powder [23], thus explaining the origin of the liquid MoSi_2_ discussed in this paper. In addition, MoSi_2_ may react with the free carbon in ZrC.

The possible reactions are as follows:

5MoSi_2_(s) + 7C(s) = Mo_5_Si_3_(s) + 7SiC(s)       T = 1850 °C, ∆G = −133.16 KJ/mol (3)

5Zr(s) + Mo_5_Si_3_(s) = Zr_5_Si_3_(s) + 5Mo(s)       T = 1850 °C, ∆G = −171.31 KJ/mol (4)

Zr_5_Si_3_(s) + MoSi_2_(s) = 5ZrSi(s) + Mo(s)       T = 1850 °C, ∆G = −46.53 KJ/mol (5)

The above reaction represents the origin of silicon carbide in this study. Concurrently, it also generated low-melting-point silicon zirconium compounds (liquid phase at the sintering temperature), which further promoted grain rearrangement and mass transfer during the sintering process [24]. Therefore, incorporating MoSi_2_ transforms the solid-phase sintering of ZrC into the co-sintering of solid–liquid phases, which reduces the densification temperature and significantly improves the sintering performance.

In addition, Mo, Zr-Si compounds, and SiC were observed in the above reaction. Apart from SiC, however, no traces of other products were detected under the electron microscope. Possible explanations for this phenomenon are as follows: firstly, the low addition of MoSi_2_ resulted in a reduced yield of the product; secondly, the zirconium–silicon compounds and molybdenum that were formed may have been dissolved during the etching of the crystal surface using hydrofluoric acid. In addition, molybdenum has a high solubility (0.6–6 vol%) in zirconium carbide [25]. Molybdenum reacts with oxygen to form molybdenum oxide, which has a melting point of 795 °C and can be sublimated and volatilized at elevated temperatures [26]. As a result, no traces of generators other than silicon carbide were found.

**Figure 9:**
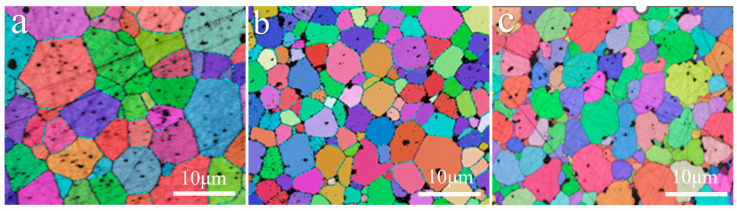
The EBSD images of the ZrC ceramics sintered at 1850 °C: (**a**) ZC0; (**b**) ZC2.5; and (**c**) ZC5.

The grain size of ZrC ceramics was significantly reduced, as shown in Figure 7d,e. The reduction in grain size can be attributed to the ZrC grain boundary at the SiC particle being bent due to anchoring, and the other parts still moving under the driving force, inhibiting the growth of ZrC grains [27,28,29]. Grain size analysis using EBSD showed that the average grain size of ZC0 was 9.08 μm. In contrast, the average grain size of ZC2.5 was 5.4 μm, and the average grain size of ZC5 was similar to that of ZC2.5. It was 5.8 μm (as shown in Figure 9). In addition, we observed that an increase in the MoSi_2_ content lead to an increase in surface defects, which was due to the incompatible interfacial bonding between the different products.

### 3.3. Effect of MoSi_2_ Content on Thermal Properties of ZrC Ceramics

**Figure 10:**
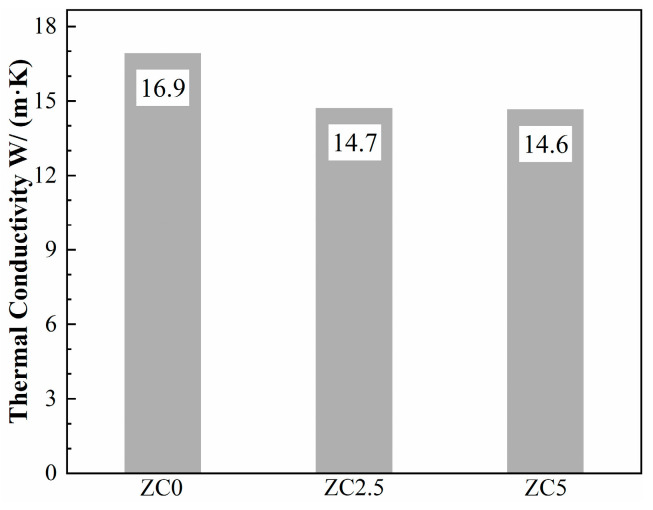
The thermal conductivity of the ZrC ceramics sintered at 1850 °C.

The room-temperature thermal conductivities of ZrC ceramics prepared with different MoSi_2_ contents are shown in Figure 10. The thermal conductivity of ZrC ceramics decreases with the increase in MoSi_2_ content, but the overall change is not significant. The room-temperature thermal conductivity of ZC0 is the largest, 16.9 W/((m∙K)), which is slightly lower than its theoretical thermal conductivity of 20.5 W/((m∙K)). The reason may be that the impurity atoms in the initial powder accumulate at the grain boundaries, or solid solution into the ZrC lattice causes lattice distortion, which, in turn, enhances its scattering of phonons, resulting in a reduction in thermal conductivity. In addition, the thermal diffusivities of ZC0, ZC2.5, and ZC5 are 7.17 mm^2^/s; 6.26 mm^2^/s; and 6.24 mm^2^/s; respectively. However, the specific heat capacities of the three samples are similar to 0.35 (J/(g∙K)). Thus, the introduction of MoSi_2_ changed the thermal diffusion coefficient of the material, which, in turn, affected the thermal conductivity. The thermal conductivity of the two-phase material can be calculated from Kingery’s law:

(6) $$\varphi_{m}=\varphi_{0}\frac{1+2V_{d}(1-\frac{\varphi_{0}}{\varphi_{d}})/\left(1+\frac{{2\varphi }_{0}}{\varphi_{d}}\right)}{1-V_{d}(1-\frac{\varphi_{0}}{\varphi_{d}})/\left(1+\frac{\varphi_{0}}{\varphi_{d}}\right)}$$

In the formula, $V_{d}$ is the volume fraction of the diffuse phase, $\varphi_{0}$ is the thermal conductivity value of the continuous phase, and $\varphi_{d}$ is the thermal conductivity value of the diffuse phase. The ZrC matrix can be counted as the continuous phase, while MoSi_2_ is the diffuse phase. The thermal conductivity values of ZrC and MoSi2 are, respectively, 20.5 W/(m∙K) and 45 W/(m∙K). The equation shows that the thermal conductivity increases with the increase in the content of MoSi_2_, but this is contrary to the conclusions we have obtained because, in addition to the influence of the intrinsic thermal conductivity of the material, it is also affected by factors such as grain size, porosity, and so on. Since the relative density and porosity of the samples are not very different, the grain size and boundary state are the keys to determining the thermal conductivity. The grain size affects the grain boundary contact area per unit volume, which, in turn, affects the thermal conductivity of the material. In addition, the state of grain boundaries also significantly affects the thermal conductivity of a sample for two main reasons: first, atoms at grain boundaries exhibit more disorder than atoms inside the crystal, resulting in lower thermal conductivity at these boundaries; second, phonons and electrons are scattered due to the presence of grain boundaries, which reduces their mean free path lengths, and, thus, the overall thermal conductivity of the material. The grain boundary thermal resistance of the material is inversely related to the grain size. ZC0 demonstrates the largest grain size among these samples, resulting in it having the lowest grain boundary thermal resistance and highest thermal conductivity.

### 3.4. Effect of MoSi_2_ Content on Mechanical Properties of ZrC Ceramics

**Figure 11:**
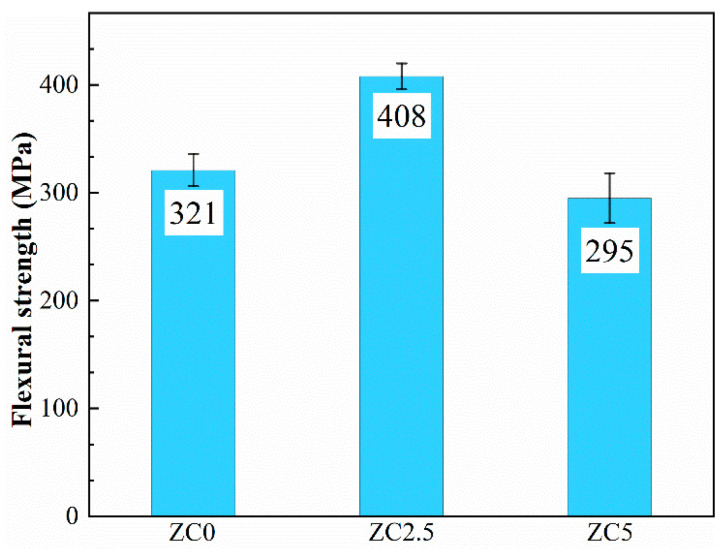
The flexural strength of the ZrC ceramics sintered at 1850 °C.

The flexural strength properties of the samples are shown in Figure 11. The general trend observed is that the flexural strength increases and then decreases with increasing MoSi_2_ content. The flexural strength of zirconium carbide (ZC0) without the addition of MoSi_2_ is only 321 ± 17 MPa. However, the sample containing 2.5 vol% MoSi_2_ (ZC2.5) shows the highest flexural strength, of up to 408 ± 12 MPa. This is much higher than the flexural strengths of the samples of pressureless-sintered ZrC ceramics reported earlier in the literature (as shown in Table 3). For ceramic materials, the modulus of elasticity, which is a porosity-sensitive mechanical property only, does not fluctuate much, only up and down to 340 ± 20 GPa, due to the high densities of all the ZrC samples. The observed variation in bending strength can be primarily attributed to grain size and porosity differences [30]. Under the premise of low porosity, according to Griffth’s formula:

(7) $$\sigma_{f}=K_{Ic}/Y\sqrt{c}$$

In the above equation, $\sigma_{f}$ is the flexural strength, $K_{Ic}$ is the fracture toughness, Y is a constant related to geometry, and c is the critical crack size in the material. Since no significant macroscopic defects were observed, and since the samples’ fracture toughness was similar and no obvious macroscopic defects were observed, the grain size was considered as the critical crack size [31]. Therefore, larger grain sizes resulted in smaller bending strengths in the samples. However, with the further increase in MoSi_2_ content, the content of the impurity phase and second phase generated by the reaction increased significantly. The second phase and impurity phase with low melting points had low intrinsic bending strength, such as the MoSi_2_ intrinsic bending strength being only 202 MPa. Therefore, the increase in the content of the second phase of low intrinsic bending strength was the main reason for the decrease in the bending strength of ZC5. Consequently, ZC2.5 exhibited remarkable resistance to bending.

**Table 3:** Comparison of flexural strength of pressureless-sintered ZrC.

Raw Materials	Temperature (°C)	Flexural Strength (MPa)	Reference
ZrC; MoSi_2_	1850	408 ± 12	This paper
ZrO_2_; C; SiC	1900	327.2 ± 16.2	[18]
ZrC; MoSi_2_	1950	272 ± 12	[16]
ZrO_2_; C	2000	308 ± 14	[32]

**Figure 12:**
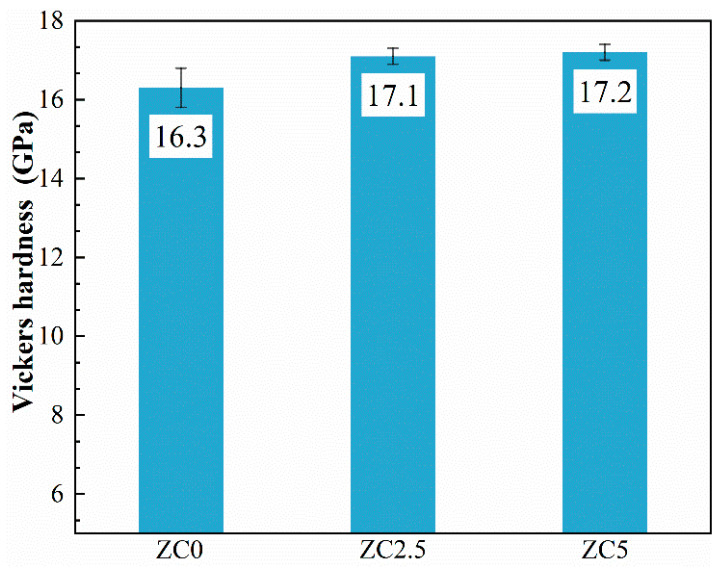
The Vickers hardness of the ZrC ceramics sintered at 1850 °C.

The Vickers hardness of the samples is shown in Figure 12. With increased MoSi_2_ content, the Vickers hardness shows an overall upward trend. The hardness values of ZC2.5 and ZC5 are similar, and ZC5 has the highest hardness value of 17.2 ± 0.2 GPa. For ceramic materials, the hardness value is influenced by the porosity and grain size of the material [33]. To completely exclude the effect of porosity and relative density on hardness, ZC0 sintered at 1800 °C and ZC2.5 sintered at 1750 °C were chosen for comparison, where ZC2.5 was 17.0 ± 0.16 GPa and ZC0 was 16.1 ± 0.3 GPa. Smaller grain size and lower porosity both contributed to higher hardness values in the ceramics, but, from the above comparisons, the main factor causing the change in hardness in this paper was grain size. In addition, the addition of MoSi_2_ had no noticeable effect on the fracture toughness of the samples, and its fracture toughness was 2.5 ± 0.4 MPa·m^1/2^.

## 4. Conclusions

Zirconium carbide (ZrC) ceramics with high relative densities were successfully fabricated via pressureless sintering. MoSi_2_, as a sintering additive, promoted the mass transfer rate during the sintering process, lowered the densification temperature, and increased the relative density of ZrC ceramics. The relative density of ZrC ceramics with 2.5 vol% MoSi_2_ was 99.1% at a sintering temperature of 1850 °C. MoSi_2_ reacted with ZrC to form silicon carbide, and its pinning effect inhibited the growth of ZrC grains, which improved the mechanical properties of ZrC ceramic materials. The flexural strength of ZrC ceramics was as high as 408 ± 12 MPa, which significantly improved the reliability of ZrC ceramics. These findings are of great significance for engineering applications.

## Figures and Tables

**Table 1:** Characteristics of the starting power.

Materials	Purity (wt%)	Mean Particle Size (μm)	Impurity (wt%)
ZrC	>99.5	0.7	Fe < 0.05%; Ti < 0.002% Si < 0.001%; Mg < 0.001%
MoSi_2_	>98.5	0.5	O < 1%, C < 0.09%, Fe < 0.02%, Ni < 0.05% W < 0.03%, Ca < 0.005%

**Table 2:** Composition design, sintering process, and characterization code of ZrC ceramics.

Identification Numbers of Samples	Compositions of Raw Materials (vol.%)		Sintering Parameters
	ZrC	MoSi_2_	
ZC0-1750	100	0	1750 °C/1 h
ZC0-1800	100	0	1800 °C/1 h
ZC0-1850	100	0	1850 °C/1 h
ZC0-1900	100	0	1900 °C/1 h
ZC0-1950	100	0	1950 °C/1 h
ZC2.5-1750	97.5	2.5	1750 °C/1 h
ZC2.5-1800	97.5	2.5	1800 °C/1 h
ZC2.5-1850	97.5	2.5	1850 °C/1 h
ZC2.5-1900	97.5	2.5	1900 °C/1 h
ZC2.5-1950	97.5	2.5	1950 °C/1 h
ZC5-1750	95	5	1750 °C/1 h
ZC5-1800	95	5	1800 °C/1 h
ZC5-1850	95	5	1850 °C/1 h
ZC5-1900	95	5	1900 °C/1 h
ZC5-1950	95	5	1950 °C/1 h

## Data Availability

Data are contained within the article.

## References

[1] Rich V. (1986). Chernobyl: Pact for nuclear accidents. Nature.

[2] Locatelli G., Mancini M., Todeschini N. (2013). Generation IV nuclear reactors: Current status and future prospects. Energy Policy.

[3] Murty K.L., Charit I. (2008). Structural materials for Gen-IV nuclear reactors: Challenges and opportunities. J. Nucl. Mater..

[4] Chant I., Murty K.L. (2010). Structural materials issues for the next generation fission reactors. JOM.

[5] Hamilton S., Jerred N.D., Scott R., Bachhav M., Yao T., Miller V.M. (2023). Diffusion study of uranium mononitride/zirconium carbide composite for space nuclear propulsion. J. Nucl. Mater..

[6] Gosset D., Dollé M., Simeone D., Baldinozzi G., Thomé L. (2008). Structural evolution of zirconium carbide under ion irradiation. J. Nucl. Mater..

[7] Cheng X.Y., Yang X., Liu M.L., Shao Y., Liu B., Liu R. (2023). Preparation of ZrC coating in TRISO fuel particles by precise transportation of solid precursor and its microstructure evolution. J. Nucl. Mater..

[8] Wei X., Song J., Liu J.X., Qin Y., Li F., Liang Y., Zhang G.J. (2021). Graphite nanoplatelets toughened zirconium carbide ceramics prepared by spark plasma sintering. Ceram. Int..

[9] Spivak I.I., Klimenko V.V. (1973). Densification kinetics in the hot pressing and recrystallization of carbides. Sov. Powder Metall. Met. Ceram..

[10] Zhao L., Jia D., Wang Y., Rao J., Yang Z., Duan X., Zhou Y. (2010). ZrC–ZrB2 matrix composites with enhanced toughness prepared by reactive hot pressing. Scr. Mater..

[11] Sciti D., Guicciardi S., Nygren M. (2008). Spark plasma sintering and mechanical behaviour of ZrC-based composites. Scr. Mater..

[12] Sciti D., Nygren M. (2008). Spark plasma sintering of ultra refractory compounds. J. Mater. Sci..

[13] Gendre M., Maître A., Trolliard G. (2010). A study of the densification mechanisms during spark plasma sintering of zirconium (oxy-)carbide powders. Acta Mater..

[14] Bulychev V.P., Andrievskii R.A., Nezhevenko L.B. (1977). The sintering of zirconium carbide. Sov. Powder Metall. Met. Ceram..

[15] Lanin A.G., Marchev E.V., Pritchin S.A. (1991). Non-isothermal sintering parameters and their influence on the structure and properties of zirconium carbide. Ceram. Int..

[16] Silvestroni L., Sciti D. (2008). Microstructure and properties of pressureless sintered ZrC-based materials. J. Mater. Res..

[17] Chantikul P., Anstis G.R., Lawn B.R., Marshall D.B. (1981). A Critical Evaluation of Indentation Techniques for Measuring Fracture Toughness: II, Strength Method. J. Am. Ceram. Soc..

[18] Zhao L., Jia D., Duan X., Yang Z., Zhou Y. (2011). Pressureless sintering of ZrC-based ceramics by enhancing powder sinterability. Int. J. Refract. Met. Hard Mater..

[19] Min-Haga E., Scott W.D. (1988). Sintering and mechanical properties of ZrC-ZrO_2_ composites. J. Mater. Sci..

[20] Gokhale A.B., Abbaschian G.J. (1991). The Mo-Si (Molybdenum-Silicon) system. J. Phase Equilibria.

[21] Fan X., Hack K., Ishigaki T. (2000). Calculated C–MoSi2 and B–Mo5Si3 pseudo-binary phase diagrams for the use in advanced materials processing. Mater. Sci. Eng. A.

[22] Pelaccio D.G., El-Genk M.S. (1994). A Review of Nuclear Thermal Propulsion Carbide Fuel Corrosion and Key Issues. Final Report [EB/OL]. https://api.semanticscholar.org/CorpusID:134572786.

[23] Gasparrini C., Rana D.S., Le Brun N., Horlait D., Markides C.N., Farnan I., Lee W.E. (2020). On the stoichiometry of zirconium carbide. Sci. Rep..

[24] Guo S., Kagawa Y., Nishimura T., Tanaka H. (2008). Pressureless sintering and physical properties of ZrB2-based composites with ZrSi2 additive. Scr. Mater..

[25] Landwehr S.E., Hilmas G.E., Fahrenholtz W.G., Talmy I.G. (2008). Processing of ZrC–Mo Cermets for High Temperature Applications, Part II: Pressureless Sintering and Mechanical Properties. J. Am. Ceram. Soc..

[26] Zhu Y.T., Shu L., Butt D.P. (2002). Kinetics and Products of Molybdenum Disilicide Powder Oxidation. J. Am. Ceram. Soc..

[27] Rezapour A., Balak Z. (2020). Fracture toughness and hardness investigation in ZrB2–SiC–ZrC composite. Mater. Chem. Phys..

[28] Fahrenholtz W.G., Hilmas G.E., Talmy I.G., Zaykoski J.A. (2007). Refractory Diborides of Zirconium and Hafnium. J. Am. Ceram. Soc..

[29] Liu J.-X., Huang X., Zhang G.-J. (2013). Pressureless Sintering of Hafnium Carbide–Silicon Carbide Ceramics. J. Am. Ceram. Soc..

[30] Neuman E.W., Fahrenholtz W.G., Hilmas G.E. (2022). Processing and mechanical properties of hot-pressed zirconium diboride—zirconium carbide ceramics. J. Eur. Ceram. Soc..

[31] Cook R.F., Lawn B.R., Fairbanks C.J. (1985). Microstructure-strength properties in ceramics. I: Effect of crack size on toughness. J. Am. Ceram. Soc..

[32] Schönfeld K., Martin H.-P., Michaelis A. (2017). Pressureless sintering of ZrC with variable stoichiometry. J. Adv. Ceram..

[33] Lee H., Speyer R.F. (2002). Hardness and Fracture Toughness of Pressureless-Sintered Boron Carbide (B4C). J. Am. Ceram. Soc..

